# A review of peripherally inserted central catheters and various types of vascular access in very small children and pediatric patients and their potential complications

**DOI:** 10.25122/jml-2020-0011

**Published:** 2021

**Authors:** Gholamreza Bahoush, Pourya Salajegheh, Ali Manafi Anari, Alireza Eshghi, Behzad Haghighi Aski

**Affiliations:** 1. Department of Pediatrics, Ali Asghar Children Hospital, Tehran, Iran (the Islamic Republic of); 2. Faculty of Medicine, Iran University of Medical Sciences, Tehran, Iran (the Islamic Republic of); 3. Neuroscience Research Center, Institute of Neuropharmacology, Kerman University of Medical Sciences, Kerman, Iran

**Keywords:** peripherally inserted central catheter, pediatric patients, complications, PICC insertion

## Abstract

Accessing the veins for blood delivery, sampling or nutrition is a critical factor in the process of care and management of pediatric patients. In this regard, the peripherally inserted central catheter (PICC) is one of the main alternatives which could be applied effectively as traditional central venous devices in neonates and adults. Due to their essential role in providing safe central venous entry, PICCs could be applied extensively in patients who are critically ill. The main aims of the present study are to review approximately all relevant publications concerning PICC procedures, any possible complications, and the most appropriate decision for preventing these complications due to their high mortality rate. We carried out a comprehensive search on PubMed, HubMed, EMBASE, MEDLINE, Science Direct, Scopus, MEDLINE, and EMBASE databases for identifying the most relevant publications related to potential complications following the application and insertion of PICCs in hospitalized children and infants. Through appropriate care of catheters, the rate of possible infectious, mechanical and thrombotic complications would decrease considerably compared to those patients who received traditional central venous catheters. However, the process of vascular access in neonatal and children is very challenging. Any delay or denying treatment due to the lack of vascular access is intolerable. In this regard, anesthesiologists must achieve extra knowledge of various vascular devices.

## INTRODUCTION

Expansion of peripherally inserted central catheters (PICCs) application has begun in the first decade of 1980. When applying long-term intravenous drug therapy, PICCs have a vital role in improving the care quality among pediatric patients due to the fact they are administered as a central venous catheter for chemotherapeutic agents and antibiotics. They also have an essential role in neonates admitted to neonatal intensive care units (NICU) for a long time and receive parenteral nutrition and drugs [1, 2]. In accordance with the worldwide database, PICCs are applied in about two and a half million people annually in acute care facilities. Because PICCs have various advantages such as ease of placement, fewer complications, shorter procedure time, a higher rate of patient satisfaction, and a more reliable form of intravenous (IV) access, PICCs are considered to be a salient tool for the management of critically ill neonate and children [3, 4].

Providing appropriate intravenous (IV) access among neonate and pediatric patients is still a significant challenge. Due to inappropriate visualization and small veins, mainly among the pediatric populations, the process of intubation could be carried out difficultly, and frequent venipunctures could be the main reason for considerable stress among neonates and children who need long-term IV infusions [5]. It should be noted that PICCs have been applied for decades among pediatric patients for improving the medical and fluid therapy outcomes during intermediate- to long-term IV infusions. Long-term vascular access is often required in neonatal patients for the delivery of medications and parenteral nutrition. However, the application of PICCs could significantly increase the success rate of insertion and simultaneously decrease the catheter-induced complications [6, 7].

Due to the improved insertion techniques, availability of new materials, and various types of new catheters, such as catheters with a lower diameter and higher volume, increased the application of PICCs for a broader range of purposes among pediatric populations. However, due to the differences in feasible catheter diameters, location and size of available veins, general activity level, immunocompetence, and underlying conditions, the comparison of infants and older children with neonates could not be adequately justified [8].

Although several benefits could be achieved from the application of PICCs, some studies have demonstrated that there are various complications that could be induced by PICC. These complications are mainly including central line-associated bloodstream infection (CLABSI) or catheter-related bloodstream infection (CRBSI), venous thrombosis, and mechanical failure (PICC line migration, obstruction, and fracture) [9]. One of the main and acute complications of PICC is an infection, with an incidence rate in the range of 16.4% to 28.8%. Moreover, the other frequent complication that could be induced by PICC is thrombosis; it is mainly present among critically ill patients [10]. The most common complications induced by PICC removal among the neonatal population include mechanical complications like thrombosis, line occlusion, intravenous infiltration, CLABSI, and life-threatening complications like pleural effusion, pericardial tamponade, and pericardial effusion [11–13].

Within their study, Pan *et al*. demonstrated that the occurrence rate of PICC-related thrombosis and mechanical complications fall between 13–91% and 35–48%, respectively [14].

Due to the serious concern about the frequent occurrence of PICC-induced complications, various studies have been carried out to prevent these kinds of difficulties. Some interventions should be carried out by the PICC team to minimize these complications, which vary based on patients' health and age. In this regard, for preventing CLABSIs, some actions such as decreasing extra manipulations, washing hands before handling the catheter, disinfecting the puncture site thoroughly, and improving the quality of available sterile techniques should be performed [15].

The application of PICC lines for long-term intravenous (IV) purposes could be considered as an appropriate supplement and/or alternative to conventional central venous lines. Recent studies have reported a considerable complication rate, which mainly includes serious bloodstream infections and venous thrombosis. However, there is not much information on the usage of this kind of catheter in the pediatric population [16]. As a consequence, the main aim of this study is to collect and review the newest literature concerning the placement methods and maintenance of PICC lines and indications among neonatal and pediatric populations for providing trustworthy knowledge and recommendations for achieving the most appropriate clinical practice. In this regard, the topic of complications associated with catheter placement is under special attention, which plays a vital role in selecting various IV devices.

## MATERIAL AND METHODS

**Figure 1:**
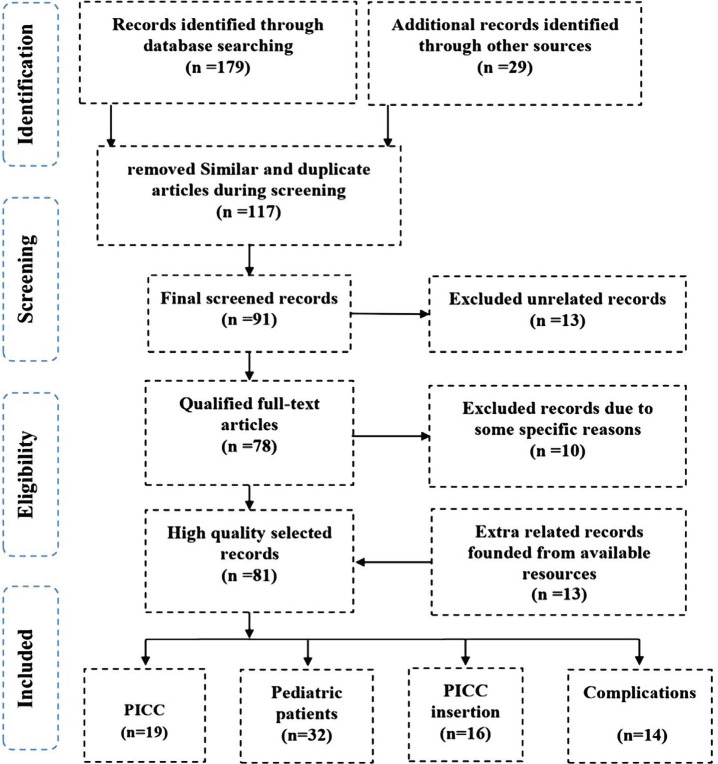
The schematic diagram of the article selection process in the current study based on the PRISMA method.

A comprehensive literature search was carried out using PubMed, HubMed, EMBASE, MEDLINE, Science Direct, Scopus, MED-LINE, and EMBASE databases using the following keywords: peripherally inserted central catheter (PICC), pediatric patients, related complications and prevention. Nearly all medical, preventive, and care publications from 2005 to 2019 were the primary focus of the search. For clarifying the results of search quality, some phrases that included PICC and pediatric and neonate patients, PICC and complications PICC and infections, as well as PICC and prevention, were applied. All found papers were evaluated for being sure about their quality. In this regard, 215 articles were found; then, the articles with high similarities were eliminated to prevent extra work. After precise screening and detailed reviewing of the articles, some other articles were deleted due to particular reasons, and a few new records were added through other available databases. Finally, the most qualified full-text articles were selected, and 81 articles were chosen as the highest quality records that should be reviewed in more detail. The schematic diagram of the selection process of articles in the present study is precisely demonstrated in Figure 1.

## RESULTS

### PICC application

At the present time, the PICC insertion procedure is performed through an aseptic technique by a dedicated team of professional physicians and well-trained nurses within a private sterile department [17]. The length of the catheter is measured from the start point of insertion to the middle point of the small cartilaginous process. The catheter insertion process is carried out through blind intravenous (IV) cannulation with the guidance of ultrasound technology. Then, the position of the catheter tip is confirmed with the help of radiography imaging techniques [18]. On the other hand, the portable ultrasound probe system is used for identifying the most appropriate vein for insertion before PICC placement. A chest x-ray is carried out to ensure the position of the catheter tip at the distal third of the superior vena cava [16], although catheter insertion could be carried out through any peripheral veins and will end up in the inferior or superior vena cava among neonates. The most common sites for insertion of the catheter include the scalp, legs, and arms. Among neonates, insertion of PICC lines could be carried out through the basilic vein and large blood axillary vessels in the upper limb, the long saphenous vein, and popliteal veins at the level of the lower extremity, and posterior auriculars and superficial temporal veins at the level of the scalp [20].

Because the suture-free technique could decrease unplanned removal and dislodgement in comparison with tape securement, it is performed for fastening the introduction site of the catheter. However, the catheter introduction site will be dressed with chlorhexidine-impregnated transparent films later. Similarly, the catheter should be flushed immediately after placement whenever used and once a week when it is not used by applying 10 ml of normal saline (NS) [21].

However, the application of NS for locking and flushing purposes is not easily accepted by PICC nurses around the world. This is mainly due to the fact that the application of heparin saline (HS) for locking and flushing of the catheter purposes is preferred by nurses in their practices [22]. The PICC size is selected based on the pediatric patient's vein diameter that would vary based on their age and mainly the pediatric patients who need total parenteral nutrition (TPN) [9]. It should be noted that for pediatric patients who are hospitalized, the dressing should be changed every week or as required in situations when there is moisture, drainage, or bleeding [23]. Manufacturing PICCs by polyurethane materials is increasingly favored due to their adequate wall strength and high flexibility. The availability of this kind of property provides the possibility of manufacturing small-sized catheters with the potential of making higher flows with a lower risk of rupture [24]. Recently, the trend of using polyurethane PICC lines dominated the market, with 70% of all PICC lines manufactured from polyurethane in the United States. This rate rose to 95% in 2017, thereby forcing silicon catheters nearly out of the market. Nowadays, more than 90% of PICCs are manufactured from polyurethane materials [25].

### The PICC insertion procedure

There is an extra demand for creating a standardized method for PICC insertion in order to intensify and support the most appropriate nursing practice to enhance the pediatric patient's welfare and safety. Special steps that could provide specific nursing policies, protocols, and procedures are presented in Table 1.

**Table 1:** Standardized steps for presenting more appropriate protocols, policies, and procedures that promote pediatric patient safety and comfort. Derived in accordance with Sona et al. [8].

Steps	The mission
**1**	The main purpose of conducting this procedure should be clarified to their parents or family members before the onset of the procedure in order to bring awareness regarding the overall steps and the logic of the procedure to make the procedure more comprehensible to the child’s family [26].
**2**	In accordance with the hospital policy, the management of various steps of mandatory procedures and equipment should be performed by highly educated personnel such as an interventional radiologist, an anesthesiologist and a specialized nurse in the operating room [9, 27, 28].
**3**	For preparing the skin effectively, chlorhexidine (an antimicrobial disinfectant) should be applied before the insertion of the catheter.On the other hand, for preventing and limiting infection, the surrounding area of the skin should be dressed with sterile drapes [29].In this regard, gloving, gowning, masking and hand hygiene are the main factors for providing care [10].
**4**	At first, the tourniquet should be placed exactly under the shoulder, the cannulation site should be rubbed with an appropriate sterile gel, and finally, for improving the access to the most appropriate vein, ultrasound guidance should be applied [9,30,31].
**5**	Under special sterile conditions, the intradermal injection of lidocaine 1% should be carried out. Under the guidance of ultrasound, the process of insertion of a thin needle should be performed through enlargement of the insertion site using a scalpel blade for leading the guidewire through the needle. If blood return is observed, the tourniquet should be loosened immediately. Finally, the needle should be taken away, and the PICC line should be placed through the vein over the guidewire to thesuperior vena cava (SVC) [31–33].
**6**	The guidewire which is fixed in the child’s body for a sustained period of time should be pulled out and then the injection cap shouldbe attached to the catheter hub.
**7**	The measuring and flushing process of the catheter with normal saline (NS) must be carried out again for returning of the blood.Moreover, the position of the catheter tip at the distal third of SVC should be confirmed by the application of chest radiography [32, 33].
**8**	Finally, the specified site of insertion of the catheter should be fixed by applying the suture-free technique and dressed by means of transparent films of chlorhexidine-impregnated dressing [34].
**9**	The overall management of pediatric patients is vital after the operation for preserving PICC patency and prevention of any possible complications. Aseptic handwashing techniques must be practiced during the overall procedure of dressing, changing, replacing of soiled dressing, medications, and administering intravenous infusions [33–35].
**10**	The vital signs and symptoms of the patient should be recorded to provide proper monitoring of the patient's condition, check the overall condition of patients, and communicate with other caregivers; in this way, adequate care is delivered, and PICC-induced complications are prevented [36].

### PICC indications among pediatric patients

When intermediate or long-term IV access is required for fluid replacement and medications, parenteral nutrition (PN), or blood sampling purposes, PICCs may be needed. The patency of peripheral intravenous catheters (PIVCs) is short, and their insertion sites often need to be changed during extended IV therapy due to exhaustion [37]. Central venous catheters (CVCs) that are placed in the subclavian or jugular veins or long-term central venous devices that are placed through surgical operations are other available options. Two of the main long-term central venous devices include implantable venous access port systems (IVAPS) and cuffed and tunneled central venous catheters. Even though other central venous devices need general anesthesia, PICC placement could be done with or without sedation [38].

On the other hand, applying other central venous devices instead of PICCs would increase the risk of serious perioperative complications like severe hematomas, air embolism, and pneumohemothorax (summarized in Table 2). Pediatric populations which undergo PICCs instead of peripheral IV catheters need one or two weeks of follow-up IV therapy [39]. Conducting intermediate-term intravenous therapy is often demanded when performing prolonged antibiotic therapy. However, applying PICCs could be more appropriate in the case of inpatients and outpatients who need therapy for up to six weeks [40]. Moreover, PICCs have a considerable role in the long-term treatment of children with oncological disorders. Consequently, PICCs could be applied for both long-term repeated infusion and blood sampling [41, 42].

**Table 2:** The comparison of peripherally inserted central catheters with various intravenous access devices. Derived in accordance with Westergaard et al. [43].

	Peripheral venous catheter (PIVC)	Peripherally inserted central catheter (PICC)	Tunneled Central Venous Catheter (TCVC)	Central venous catheter (CVC)
**Serious insertion complications**	No	Very rare	Potential	Potential
**Serious systemic complications**	No	Potential	Potential	Potential
**Insertion difficulty**	Easy	Easy	Difficult	Difficult
**Need for general anesthesia (GA)**	Rare	Sometimes	Always	Always
**Need for surgical removal**	No	No	Yes	No
**Patient compliance**	+	++	+++	+
**Patency**	Days	Weeks	Months	Weeks
**Catheter cost**	+	+++	++++	++
**Mechanical difficulties (fracture, dislodgement, occlusion)**	Often	Sometimes	Sometimes	Sometimes

### Appropriate short-term CVCs in pediatric patients

These plastic tubes are useful devices for short-term therapy or emergencies in which their expected dwell time is less than seven days. The configurations of these kinds of devices are available in up to five lumens. Electrical devices which could be used for injection are widely available. The application of this kind of device among adults is very common, but their application among children would increase the complication rates and results in lower success rates. However, due to the higher sensitivity of the pediatric body, it is advised to use ultrasound for more appropriate guidance of the insertion of catheters [44, 45].

Based on the advice of Scott *et al*., the catheter size could be determined by the vein size and requirements of the therapeutic procedure, as with PICCs [46]. Based on the standardized guidance, size 4–5 Fr catheters are usually appropriate for infants younger than six months, size 5 Fr for pediatric patients aged between six months and five years, and size 7 Fr for those patients who are older than five years [47]. Even though special formulas based on surface-landmark, weight, and height have been developed for guiding the most appropriate length of catheter insertion, another method is used in practice: for children whose weight is lower than 15 kg, this length is determined to be 5 cm lines, for children in the weight range of 16–40 kg, the length is 8 cm lines, and for those higher than 40 kg, this length is about 13 cm lines. In this regard, the most appropriate position of the line tip must be confirmed by radiography imaging [48].

### PICC complications among children

Due to the inconsistent studies among pediatric populations, the association between catheter-tip location and PICC complications is not clearly well understood. For instance, some studies have demonstrated that the insertion of PICCs in non-central veins could provide more safe and reliable IV access. Other studies found that terminating PICCs through non-central venous locations would increase the risk of possible complications [49]. Consequently, the comparison of these studies is not easy because their definitions of central veins are inconsistent. The possibility of an inappropriate combination of various factors such as vessel size, turbulence, endothelial injury, and blood flow rate would increase mechanical complications, mainly in non-central PICCs [43]. Moreover, based on the studies carried out by Ketan *et al*., non-central catheters would independently increase the non-infectious complications [50]. When a CVC is positioned within the superior vena cava, the catheter tip lies in the parallel position to the vessel wall. Consequently, the process of diluting the solution will happen rapidly. Due to the possibility of vessel tortuosity, venous valves, and venospasm, the process of advancement of PICCs to the SVC will not always happen [50]. When the catheter tip is placed in a non-central location, complications may happen; the connection of the tip with the vessel irritates and disrupts the endothelial layer of the cell, triggering blood clotting and exposing the basement membrane. The process of insertion of non-centrally located PICCs should be carried out carefully because catheter removal is associated with higher rates of complication [51].

Another risk factor for PICC complications is catheter dwell time, so that shorter or longer dwell times could increase the complication rates [52]. In accordance with the studies carried out by Ketan *et al*., there is a complex interaction between catheter dwell time and the risk of PICC complications [50]. However, the risk of infectious complications is increased during the first few weeks and the risk of non-infectious complications is decreased during the first few weeks [50, 53].

As an influential factor, age may confuse the association of tip location and PICC complication because promoting PICCs among pediatric populations may be more challenging. However, these challenges may lead to unintentional non-central PICCs within this sensitive age group. Placement of PICCs within the lower extremities, neck, and head could increase the risk of unavoidable removal of complications [54]. Moreover, modifying other critical variables such as age, intensive care unit (ICU) exposure, PICC placement main indication, insertion site, the location of the catheter tip, and catheter dwell time would not be notable risk factors for complications. Regarding the potential of pediatric populations for being affected by various complications associated with PICC insertion, it should be noted that younger ages are more frequently affected than other age groups. On the other hand, the possibility of PICC insertion in the lower extremities, neck, and head is higher than other body extremities in pediatric patients, which may confound the comprehension of this association [55].

### Special considerations regarding contraindications

There are not too many contraindications for PICC placement. Some damages like radiation, burns, and infection at the insertion site could make the process of catheter securement complicated and may increase the overall risk of bacteremia or catheter colonization [43]. Localized edema may decrease the proper venous visibility and also disrupt the insertion completion. The catheter placement process may be barricaded by thrombosed, damaged and small vessels induced from the previous process of catheter insertions or frequent cannulation attempts. It should be noted that consecutive PICC insertions further increase the access difficulty [56]. Additionally, stenosis, central thrombosis, and congenital venous anomalies of the superior vena cava (SVC) may prevent the advancement of the catheter to the correct aimed position. Special considerations must be carried out in pediatric patients who suffer from end-stage kidney disease and/or chronic kidney disease. Among these patients, for prioritizing the vein's preservation for setting up an arteriovenous fistula for dialysis porpuses, other alternatives must also be considered [38, 57].

### Insertion of the PICC line

PICC insertion must be performed by a group of trained staff, including pediatricians, interventional radiologists, specialized IV nurses and anesthesiologists. This procedure could be carried out in various standardized settings such as the operating room and/or a specialized set of angiography [43, 51]. The antecubital fossa veins could be detected through palpation or visually. For veins that are deeper, the visualization process could be carried out by very high-resolution ultrasound (VHRUS) [58, 59].

Through assisted visualization, the insertion success would be significantly improved to 90–100% [41]. However, the main strategy for the use of ultrasound among the pediatric population must reflect the local organization, resources, and requirements of the patients. It should be noted that the use of ultrasound is preferred in comparison with other available imaging techniques like fluoroscopic venography. The main reasons for this popularity are simplicity in learning, high transportability, and the capability of providing more appropriate visualization of veins and adjacent structures [60]. However, ultrasonic compression probes of the vein may barricade the advancement and puncture of the guidewire. Although fluoroscopy could provide the most appropriate visualization of the veins, such as collaterals or occlusions, it is limited to angiography and needs increased exposure to radiation, intravenous contrast, and an available peripheral intravenous catheter [61].

### Enhancing patient's comfort during PICC insertion

For enhancing patient comfort, optimizing the insertion arm positioning, and even keeping it in place, the majority of children require sedation. Although there are several sedation protocols for pediatric populations, the analog-sedative strategy should be individualized [59]. However, the majority of sedation protocols are performed by means of spontaneous breathing with oxygen therapy through a laryngeal mask or using the nasal route. In children who are older than 12 years, PICC insertion could be performed by local anesthesia (LA) alone. The available options for LA include applying lignocaine for local infiltration analgesia and/or applying an appropriate local anesthetic cream on the suitable veins. Other children could be relaxed through the aspiration of nitrous oxide (50%) or midazolam premedication during the procedure [62, 63]. Table 3 presents the available strategies based on childrens' age.

**Table 3:** Various applicable strategies for enhancing the patient's comfort during PICC insertion based on their age. Derived in accordance with Westergaard et al. [43] and Vineet et al. [64].

Age group	Sedation strategy
**Younger than 6–8 years**	For this age group, it is recommended to use general anesthesia to achieve the most appropriate outcomes which gives more comfort to the patients during PICC placement.
**6–8 years**	Due to the fact that the patients within this group are older and more capable of tolerating the possible complications of PICC placement, the application of nitrous oxide, local anesthesia, and premedication is enough and there is no need for general anesthesia.
**Older than 6–8 years**	Administering premedication for achieving local anesthesia is considered to be an effective method within this age group.

### Various types of vascular access in neonates

**Figure 2:**
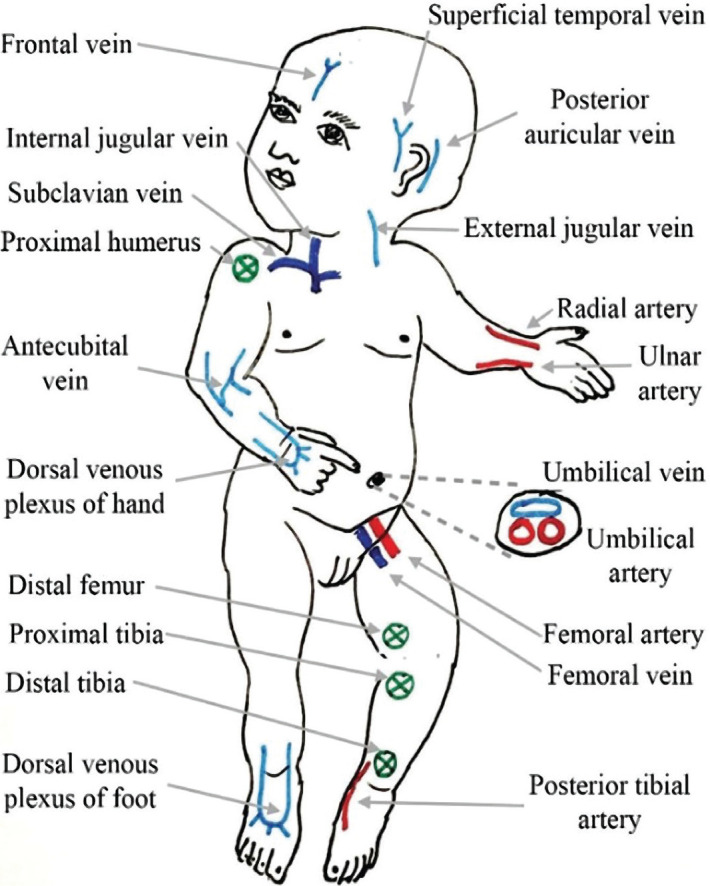
The location of the most common vascular access sites for peripheral venous catheter (light blue) central venous catheter (dark blue), arterial access (red) and intraosseous infusion (green). Derived in accordance with Vibhavari et al. [65].

As shown in Table 4, various methods of vascular access among children, their advantages and disadvantages, as well as the catheter sizes, are available. Additionally, the most appropriate sites for PICC insertion which provide suitable vascular access among children are shown in Figure 2 [65].

**Table 4:** Application of various vascular catheters in pediatric patients. Derived in accordance with Vibhavari et al. [65].

Type of catheter	Insertion site	Catheter size		Dwell time (Term)	Advantages	Disadvantages
	Leg and hand dorsum, veins of the scalp, great saphenous vein, external jugular vein, antecubital	Neonates	24 G		Economical, uncomplicated and with fewer complications	Inability to draw blood, short term usage
**Peripheral venous catheter**		Infants	22 G	Up to five days (Short)		
		Children	20 G			
**Central line midline catheters**	The forearm and arm deep veins	Neonates	24 G	Up to 2 weeks(Intermediate)	No need for radiological confirmation; inserted easily and with longerdwell time	Can not be used to draw blood; only solutions which are peripherally compatible canbe applied
		Infants	22 G			
		Children	20 G			
		Neonates	1 Fr			Requires experienced specialist, need sradiological confirmation, requires training of patients for suitable device care
	Superficial basilic and cephalic vein, brachial deep vein(The catheter size is smaller than one-third of the vein diameter)	Infants	2 Fr	Few weeks to a few months and in viable catheters up to one year(Intermediate)	Patients are more comfortable and could be sent home; the possibility of blood drawing	
**PICC**		Small children	3 Fr			
		Old children				
			4 Fr			
**Non-tunneled central line**	Femoral veins, subclavian artery and internal jugular vein	Neonates	3 Fr	1 to 2 weeks(Short)	Irritant chemotherapy agents, hyperosmolar hyperglycemic state, central venous pressure monitoring, and multiple lumens	Due to the higher risk of being affected by infection, their usage duration is limited and patients can not be sent home
		Infants	4 Fr			
		Small children	5 Fr			
		Old children	7 Fr			

### Peripheral venous catheter

One of the most popular and applicable vascular access devices is a peripheral venous catheter for which blood vessels within the dorsal venous plexus of the hand should be selected. For conducting the most appropriate cannulation among children, the medical specialist must be experienced enough in detecting venous anatomy and common insertion sites (Figure 2). Because veins of the lower extremity may cause immobilization of the child, these should not be selected as insertion sites. On the other hand, during the process of accessing antecubital fossa veins, extra care should be taken to avoid inadvertent arterial cannulation [65]. If unexpected intraoperative hemorrhage happens, the superficial external jugular vein could be selected as an alternative choice. Other available challenges of cannulating related to this vein are the shallow angle of penetration, loose skin, and collapsed vein. When appropriate access to the peripheral venous catheter among very small children and neonatal is not available, the superficial veins of the scalp could be utilized [66].

When the selection of a larger cannula is possible, it is recommended to use a smaller size of the peripheral cannula to achieve the medical purposes, except for unstable patients and emergencies. For preventing vein rolling, the skin should be stretched, and the veins should be stabilized. The entrance angle of the catheter is in the range of 10–25° for yielding more comfort until the backflow is visible. After that, the insertion angle could be dropped more until the catheter can be threaded in the vein. Moreover, extra care should be carried out to avoid more pressure injuries and ischemia [67].

### Central line midline catheters

A midline catheter is a peripheral venous catheter that could be used appropriately in prolonged antibiotic treatments. Under ultrasound guidance, the midline catheter with a length of 6–12 cm could be inserted within the arm's deep veins. On the other hand, a longer peripheral venous catheter could be placed in the veins of the mid-arm. Despite the fact this can not be utilized currently, in the situation when a single venous cannula could be enough during the care period, the application of this catheter could provide easy venous access in children undergoing surgery [68].

### PICC line access

These are used as intermediate-term venous catheters, which could be inserted in one of the deep arm veins such as cephalic, brachial, or basilic while their tip is placed in the junction of the right atrium and superior vena cava. In children who are older for catheter placement, the Seldinger technique powered by ultrasound imaging by means of the sheath over the dilator is recommended. The process of cannulation among neonates could be carried out by applying the sheath over the needle apparatus. Moreover, when the catheter size is larger than 3 Fr, PICCs could be used as central lines for blood sampling purposes [69]. On the other hand, for tolerating the higher pressures of computed tomography (CT) contrast infusion, power-injectable PICCs are developed to be used and are the best choice in oncological patients who would need frequent scans [70].

### Non-tunneled central line

Some of the most common indications for central venous catheters are the application of vasopressors, poor peripheral venous access, chemotherapy, and parenteral nutrition (PN). Non-tunneled central lines are contraindicated mainly in platelet disorders, severe coagulation, and local infections [65].

### Central venous access in small children

PICCs are mainly available in various configurations of single-, double-, and triple-lumen and in various sizes ranging from 28 G catheters to 7 Fr triple-lumen catheters for being applied among premature neonates. Based on a general convention, multi-lumen catheters are described by French (Fr) size and single-lumen catheters by gauge, as shown in Table 5 [46]. The appropriate size of PICC is specified through the main size of accessible veins and the main requirements of the therapy. The PICC size can not be determined based on the patient's age. Despite the fact that smaller catheters with fewer lumens would cause the fewest complications, the possibility of blockage of very small catheters is higher. When PICCs are required for blood sampling, their required size is at least 3 Fr [71]. IV infusions could be carried out by PICCs, while in most cases, their dwell time is less than two months and seldom more than six months. PICCs that are known as power-injectable are mainly capable of permitting intravenous contrast administration during CT scanning [72].

**Table 5:** The French gauge diameters and the sizes of the cannula. Derived in accordance with Scott et al. [46].

French gauge	External Diameter (mm)	Common intravenous cannula needle gauge	External Diameter (mm)
**-**	**-**	**24**	**0.7**
**-**	**-**	**22**	**0.9**
**3**	**1.0**	**20**	**1.1**
**4**	**1.34**	**18**	**1.3**
**-**	**-**	**17**	**1.5**
**5**	**1.67**	**16**	**1.7**
**6**	**2.0**	**14**	**2.1**
**7**	**2.3**	**-**	**-**

The sizes of intravenous cannula and French gauge equivalents. In neonates and very small children, special care should be carried out for avoiding the insertion of devices that would cause complete obstruction and would not allow blood to flow past it.

The most appropriate site for PICC insertion is the large superficial basilic vein placed above the elbow where the cephalic vein makes a small angle at its connection point with the subclavian vein, and that would be the main cause of blocking the entrance to the central-chest vasculature [72]. However, to avoid the median nerve, the brachial vein should be used carefully. Moreover, in non-ambulant children, the great saphenous vein (GSV) could be used as an alternative. In neonates, PICCs will progress from their peripheral site of insertion up to the time when the catheter tip lies either at the cavo-atrial junction or in the distal third of the superior vena cava. On the other hand, catheters that are inserted through the lower limb will be terminated in the inferior vena cava. During the insertion process, the catheter position should be confirmed by the application of post-procedure chest X-ray or fluoroscopy in the situation when surface landmarks are applied for guiding insertion depth [73].

It should be noted that ultrasound imaging could be applied for eliminating jugular malposition before radiography. Moreover, PICC insertion at the level of the upper limb in neonates and small children with any small movement of the arm will move by an average of 2.2 rib spaces. Consequently, these PICCs would not always remain in their optimal position [73]. Moreover, as a general suggestion, it should be noted that, during the insertion procedure of PICCs, the line must be fixed in a way that the tip placement is optimal even when the child's arm is placed comfortably in its natural position [73]. Additionally, the possibility of malposition of the catheter tip in children who are younger than 6 is higher than in older children and adults [74]. However, some of the main advantages of PICC insertion among neonates and small children are that they could be placed and removed without general anesthesia and would also cause the lowest complication rate compared to other central venous access devices. On the other hand, it should be noted that the permissible duration of this kind of therapy is mostly in the range of 10 to 60 days. Moreover, a device that could be used alternatively is a midline catheter whose length is longer than a peripheral cannula but shorter than the main PICC line. For achieving a larger portion of the vein with higher blood flow, this alternative device could be threaded proximally and inserted peripherally [72–74].

### Selection of appropriate catheter

The materials used in PICC are correlated with the occurrence of a variety of infections. For example, a catheter made of silicone is associated with a higher risk of infection and microorganism colonization than that made of polyurethane. The shape of an absolute catheter is similar to a single lumen polyurethane catheter with a high volume and small diameter [23]. The use of polyethylene in the manufacture of PICC provides relatively greater wall strength and allows for producing high-flow catheters with greater inner lumina while small in size. Although there seems to be no difference in complications after insertion between polyurethane and silicone catheters, there are no clinical studies comparing these two materials [64].

Although not proven in a randomized study, a large-sized PICC may increase the risk of venous thrombosis and occlusion. A PICC with a diameter of more than 5F increases the rate of UEDVT [9, 75]. In addition, the use of single-lumen PICCs with a smaller gauge is associated with lower complication rates (17.2/1000) compared with using double-lumen PICCs (30.8/1000). Liem *et al*. found a significant relationship between the diameter of PICC and thrombosis; the authors reported thrombosis rates of 1%, 6.6% and 9.8% for PICC diameters of 4F (French catheter scale), 5F and 6F, respectively [4]. They found no association between thrombosis and 3F PICCs. Large PICCs may increase the thrombosis risk, while using small catheters may produce mechanical problems [76].

AS shown in Table 6, the selection of PICC size should be based on the children's age and the vein dimensions. The most appropriate catheter is a single-lumen, high-volume with a low-diameter made of polyurethane [77].

**Table 6:** The process of selecting the most appropriate size of the catheter. Derived in accordance with Matjaz et al. [78] and Vaishali et al. [79].

Patients age group	The proposed size of the catheter (mm)
**Younger than 12 months**	In the range of 2–3
**1–6 years**	In the range of 3–4
**6–10 years**	Exactly 4
**Older than 10 years**	In the range of 4–5

### Possible complications

The most common complications related to PICC are venous thrombosis, phlebitis, local or systemic infections, and mechanical problems such as catheter leakage or breakage, occlusions, and incidental dislodgement. In pediatric populations, the rate of complication is low overall, and based on the clinical setting and type of population studied, it has been reported to vary from 1.11 to 19.3 per 1000 days of catheter [40, 41, 80]. Improper movement or replacement of PICC and catheter removal are two major causes for the most common complications among children below 5 years old. Antimicrobial treatment may be applied to treat these complications [40]. The results of available non-randomized studies show that although PICCs may result in more mechanical problems such as occlusion or incidental displacement compared with traditional CVCs, the occurrence of serious complications such as CRBSI or deep venous thrombosis (DVT) is the same or even less common. The use of PICCs may result in a considerably higher risk of DVT compared with long-term central venous devices. However, catheter occlusion is less common when using this method [81]. The overall factors that could affect PICC placement include the caregiver factor, as well as minor and major complications described precisely in Table 7.

**Table 7:** Classification of various complications during PICC placement. Derived in accordance with Sona et al. [8].

Possible complications		Main causes	Consequences
**Caregiver factor**		Mainly due to the lack of adequate practice and knowledge of the healthcare staff but also poor hand hygiene regarding PICC insertion; the appropriate technique of patient care would lead to different complications.	May cause various intolerable complications including infections which could be induced from the moment of PICC placement
**Minor complications**		Minor complications include feeling local, phlebitis of a catheterized vein, slow withdrawal of blood that could be managed by applying secondary treatment. These complications do not require any hospitalization. Due to the lack of appropriate minor treatments or conservative management, these complications can not be managed properly.	Redness, inflammation of tunica intima of the veins, severe local pain induced by inflammation
**Major complications**	**Infective complication**	These kinds of complications could be caused by early removal of the PICC line. However, different factors could cause PICC-associated complications that should be managed and prevented appropriately.	Central line bloodstream infection (CLABSI)
	**Thrombosis formation**	These kinds of complications could be caused by early removal of the PICC line. Patients who are critically ill have a higher chance of being affected via PICC-related venous thrombosis	PICC-related venous thrombosis, deep vein thrombosis (DVT) and vascular thromboembolism
	**Mechanical failure**	These kinds of complications could be caused by early removal of the PICC line. While mechanical catheter complications are not life-threatening, they may hinder the treatment process and require PICC removal or replacement. Catheter malposition could cause thrombosis. Moreover, the lack of training and knowledge of nurses about mechanical complications may increase the rate of possible complications.	Inner lumen occlusions, catheter-related occlusion complications, catheter-related obstruction, coughing and vomiting (in critically ill patients), DVT and infection

## CONCLUSIONS

There is a large body of studies on PICCs in the pediatric population. However, most of these are observational studies in which there are a few randomized controlled trials. Due to the variations in study designs and populations, it is difficult to make a comparison between these studies. Catheter patency and the incidence of complications are largely affected by the clinical setting, child immuno-competence, and use patterns.

Nevertheless, PICCs may be used as a safe option for central venous access in children either in the intermediate- or long-term. New types of PICC may facilitate broader indications and provide longer dwell times. They can be utilized either in outpatients or hospital settings. PICC placement can be learned easily, usually requires light sedation and/or local anesthesia, and seldom causes serious perioperative risks. The risk of serious complications in the long-term seems to be low so that it is comparable with that of traditional CVCs. However, the use of implantable or tunneled devices may provide safer outcomes in the long-term.

According to the above-mentioned considerations, the use of PICCs may have some benefits in the following settings:

- Short- to medium-term IV access in children receiving IV therapy (antibiotics, frequent blood sampling, total parenteral nutrition) from 4–5 days up to a few weeks;
- Long-term access to the central veins as an alternative to traditional devices (implantable port systems or TCVC) in patients with severe coagulopathy or those with contraindications to GA, such as patients with remarkable comorbidities;
- Temporary access to the central veins in oncology patients requiring the injection of toxic medications (for example, children with mediastinal or cervical pathology) until a long-term device is available.

Application of assisted visualization and having well-educated staff could improve the insertion success rate and reduce the complications of PICCs. Ultrasound is a promising procedure to use in the future. Meanwhile, efforts have been made to develop new methods that could reduce the risk of infected or occluded catheters. However, more controlled and randomized clinical studies are required to evaluate further the application of PICCs in the care of infants and children.
